# The Middlesex Hospital Reports

**Published:** 1916-08-12

**Authors:** 


### The Middlesex Hospital Reports.

In issuing the annual reports of the Middlesex Hospital and of the Cancer Charity, the respective committees have evidently been uninfluenced by what has been said as to the desirability of abbreviation in these expensive times. We are not going to reopen a highly controversial subject and remain content to make the timid suggestion that the thirty-five pages of "short history" might well have been held over for the present, and the authorities left satisfied that the history of the charity is written all over the splendid institutions in Mortimer Street and Nassau Street. The 350 beds of the hospital were used for 5,410 patients, 356 of whom were wounded soldiers. The total is not so high as in 1914, as two wards have been closed during reconstruction work. The attendances in the out-patient department were 155,211.

The Convalescent Home at Clacton-on-Sea has been used exclusively for sick and wounded soldiers since the beginning of the war. The deficit on this branch, £5,434, was made up out of hospital funds, and there does not appear to be any set-off in the way of a War Office contribution for maintenance, neither do any of the patients of the hospital, in or out, contribute anything towards the cost of maintenance, etc. Despite the receipt of £16,270 in legacies, the hospital had an adverse balance of £3,822 at the end of the year. We note that there is a mortgage on the Convalescent Home of £20,000, the interest on which was £600 in 1915. The heavy responsibility of remaining in debt while so much is given away which could be, and in the opinion of many should be, partly paid for by or on behalf of the patients is one which, we suppose, is borne and understood by the governors.

On the expenditure side we learn that on the outbreak of the war a large stock of dressings and bandages, instruments and appliances, was acquired, and the justification of that step is proved by the reduction of expenditure incurred under these headings in the previous year. Heavy prices had to be paid for steam-coal, but a saving was effected in the consumption of house-coal. The cost of each patient per week for provisions has risen from 9s. 9d. to 11s. 6d., but the expenditure under "Surgery and Dispensary" has fallen by nearly one-half, the latter fact being caused by the large stocks previously mentioned, which are in no way valued under the system of accounts now generally used, and which evidently, when drawn up, did not provide for the possibility of such important purchases for future use, the consequence in this case being that the decrease in cost is entirely fallacious.

The Cancer Charity, which issues a separate report, with its own "short history" of twenty-eight pages, maintains ninety-two beds in the cancer wing of the hospital; 483 patients occupied these beds during 1915. The total income, including £418 legacies, was £5,389, and the total expenditure £5,683. The Cancer Investigation Fund had an income of £6,427, but only spent £2,103 owing to a great portion of the work having been brought to a standstill because of the absence of a large part of the staff, who are rendering military service.

